# Reply to de Reuver et al. Comment on “Grivas et al. Morphology, Development and Deformation of the Spine in Mild and Moderate Scoliosis: Are Changes in the Spine Primary or Secondary? *J. Clin. Med.* 2021, *10*, 5901”

**DOI:** 10.3390/jcm11072049

**Published:** 2022-04-06

**Authors:** Theodoros B. Grivas, George Vynichakis, Michail Chandrinos, Christina Mazioti, Despina Papagianni, Aristea Mamzeri, Constantinos Mihas

**Affiliations:** 1 Department of Orthopedics & Traumatology, “Tzaneio” General Hospital of Piraeus, 185 36 Piraeus, Greece; vini_gio@windowslive.com (G.V.); chandrinosmichail@gmail.com (M.C.); 2 Health Visitor, “Tzaneio” General Hospital of Piraeus, 185 36 Piraeus, Greece; maziotix@gmail.com; 3 School Nurse—Health Visitor, Special Primary School of Rafina, 190 09 Rafina, Greece; papdes2009@hotmail.com; 4 Health Visitor, TOMY Attica Square, 104 45 Athens, Greece; mamzeri_aristea@hotmail.com; 5 Department of Internal Medicine, Kymi General Hospital—Health Centre, 340 03 Kymi, Greece; gas521@yahoo.co.uk

With great interest we have read the “Letter to the editor by de Reuve et al. concerning “Morphology, Development and Deformation of the Spine in Mild and Moderate Scoliosis: Are Changes in the Spine Primary or Secondary?” by Grivas et al. [1]”.

We appreciate their compliments on our work and we would like to thank them for their kind words [2]. Moreover, we should thank the authors who have studied the publication so thoroughly, providing us with a great opportunity to controvert the arguments put forward by a group of colleagues world-renowned for their studies on the aetiology of idiopathic scoliosis. The authors of the letter state that “The data presented in this study contribute to the discussion”, and they claim, “however, we believe it should be interpreted with caution since there are substantial methodological limitations, and these results should not seek to provide a definitive answer”. However, the comments by de Reuve, et al. generally apply to scoliosis cases that are more severe than the subjects examined in our series.

Furthermore, it would be useful to have a look at the definitions of the severity of scoliosis. There is not full agreement on what is mild and moderate idiopathic scoliosis. Mild idiopathic scoliosis is characterized by a Cobb angle of more than 10 and less than 30 degrees [3], of more than 10 but less than 25 degrees [4], and of more than 10 but less than 20 degrees [5]. Moderate IS is characterized by a Cobb angle of 25–40 degrees, which is indicated for non-operative treatment [6,7], and a Cobb angle greater than 21 to 35 degrees [5]. We consider as mild curves those with a Cobb angle of greater than 10 but less than 20 degrees and as moderate those with a Cobb angle of greater than 21 to 35–40 degrees. In our material, only one patient had a Cobble angle greater than 40 degrees, which is practically not affecting the mean Cobb angle.

In these curves, especially in the mild ones, the rotation of the apical vertebrae is not large [8,9]; this morphology is very important for the measurements of a true sagittal profile, which is minimally affected. This fact results in more reliable contacted measurements, and it is very important for our study.

Our response point by point to authors’ comments is presented below.

In comment number 1, the authors of the letter notice that “the study is cross-sectional and without radiographic sagittal measurements”. This research is indeed a cross-sectional one and not a longitudinal study. It could not have been a longitudinal design because we studied mild and moderate scoliosis, and it is not an attempt to establish the definite causality of IS. All these children were presented in the scoliosis clinic and diagnosed with IS for the first time; as a result, they had not had any previous imaging examination for IS. Moreover, we only used the data of diagnosis at presentation, as this was the aim of the study. Therefore, we did not aim to conduct a longitudinal study.

The letter’s authors state that “the study was done without radiographic sagittal measurements”. We do have lateral radiographs for all the IS children included in the study because this imaging is asked for, based on our protocol, during the first examination and assessment. However, the sagittal profile using surface topography (ST) is correlated to the sagittal profile to radiographic imaging with a strong correlation coefficient, not only for the surface apparatus we utilized but also for other systems of ST as well [10,11,12,13,14,15]. Therefore, for this reason, it was not deemed necessary to study the lateral radiographs of IS children. Consequently, the results of the study were not affected.

Moreover, the reference of Deacon et al. 1984 stating “that the apical deformation in IS is characterized by apical lordosis and alters the regional and global sagittal profile”, which is quite true, cannot be used for mild or moderate IS. All of Deacon’s scoliotic spines are severely deformed up to 184 Cobb degrees, except for one of 18 degrees, with a significant/severe rotation, which is not the case, as mentioned above, for mild or moderate IS. Mild IS children were analyzed in the study by Grivas et al. [16,17]; thus, a comparison to severe scoliosis is not advisable.

At this point, we are given the opportunity to reiterate and emphasize our view, which is also expressed in our publication, that is “at initiating and mild scoliosis, the patho-biomechanics are probably dissimilar from the biomechanics when the curve is severe”. Furthermore, we consider that at initiating and mild IS cases, genetics, epigenetics, and biology have the dominant/protagonistic aetiological role; however, we should not overlook the non-protagonistic role of patho-biomechanics, which later become dominant for progressive IS.

In comment number 2 in the letter, the authors claim: “A longer posteriorly inclined segment was previously shown to be associated with thoracic scoliosis, but not necessarily with (thoraco)lumbar scoliosis. For the latter in fact, a shorter but more steep posteriorly inclined segment with a more distal kyphotic apex may be much more relevant. [5,6]” We would like to note that in the study, we included no children with lumbar IS curve, as was the case in Schlösser et al. 2014 [16]; this publication did not include any thoracolumbar curve, either. Therefore, the comment on the morphology of lumbar curves is not applicable to our study.

In comment number 3 in the letter, the authors claim: “The type of power analysis and whether the test was one or two-sided is not mentioned”.

It should be stated that it was an a priori, one-sided power analysis. The types of power analyses and the selection of the margins were both based on our previously unpublished data, due to lack of sufficient published results for this specific marker (ratio of distances). Although we acknowledge the fact that, in controls, the mean difference proved lower than the expected one, it should be noticed that the main comparison of interest was between scoliosis patients and controls, rather than a gender-based comparison. However, a larger sample size would make the margin lower and thus make the whole study more robust to gender and other potentially confounding effects.

In comment number 4 in the letter, the authors claim: “the age distribution of the controls and scoliosis series do not match and they include juveniles as well as adolescents”.

In Group A (IS), the mean age of the girls was 12.4 (range: 8.4–14.3 years). The mean age of the boys was 15.3 (range:14.1–16.4 years). In Group B (Controls), the mean age of the girls was 12.2 (range: 9.3–15.2 years). The mean age of the boys was 13 (range: 9.9–15.6 years).

It is stated by the authors of the letter that “The mean VP-KA/VP-LA ratio in the controls was higher in girls, representing a relatively lower kyphotic apex than boys, which indicates a sex difference. Therefore, the scoliosis group which mostly consisted of girls, is biased to have a higher ratio. Interestingly, the ratio was still lower in the scoliosis group compared to controls, although not significant. This is important, since a trend towards a smaller VP-KA/VP-LA ratio (i.e., higher kyphotic apex) in idiopathic scoliosis, probably corresponds to a relatively longer posteriorly inclined segment, which is opposite to the authors conclusion but in line with other hypotheses in the literature [5]”. This argument is rather weak and of no use because no matter the non-statistical variations of VP-KA/VP-LA ratio among the groups, the main finding—which was not emphasized and taken into consideration by the authors of the letter—is that all of these ratios were not correlated to Cobb angle and the truncal asymmetry measured using the scoliometer. In other words, in the studied group suffering mild and moderate IS, the sagittal profile is not associated with coronal and transverse plane deformity.

In comment number 5, the authors claim, “Besides comparing the two groups, the authors demonstrate that the VP-KA/VP-LA ratio did not significantly correlate to the scoliometer trunk asymmetry measurements (r = 0.211, *p* = 0.416). Therefore, the authors conclude that the hypothesis of a larger posteriorly inclined spinal segment being a primary etiological factor for idiopathic scoliosis is not confirmed. This study was not powered for this analysis, possibly introducing a type II error”.

As far as the power analysis concerns the introduction of a type II error, it is plausible given the fact that the power analysis in our study was—as usual—performed for the purposes of the main comparison. However, the large deviation from a significance level close to 0.05 increases the probability of a real correlation.

Additionally, the authors of the letter mention the following: “But most importantly, the correlation with curve severity is not relevant for the sagittal spinal alignment, since it has been hypothesized to influence scoliosis risk, not severity. Scoliosis severity is probably mostly correlated to time from onset, not the sagittal profile”.

We did not aim to study severe curves or the prediction of which curve will become severe.

In comment number 6, the authors write: “Throughout the manuscript, the authors interchange the terms ‘posteriorly inclined segment’ and ‘hypokyphosis’, however, we would like to emphasize that these are distinct”.

The terms ‘posteriorly inclined segment’ and ‘hypokyphosis’ in a linguistic sense are completely distinct, but in IS, they are anatomically considered strictly interconnected and justifiably may be used both describing the same entity. The reason for this consideration may be described below.

When the decrease in the normal spinal thoracic kyphosis angle (T4–T12 or T1–T12) changes below a cutoff point, then this kyphosis is termed hypokyphosis. The normal kyphotic angle can only be decreased if the kyphotic apex is placed more cephalad, and then the posteriorly inclined segment of the kyphosis is increased. This can only occur if the number of the so-called “declived vertebrae”, in other words, the number of the posterior inclined vertebrae—comprising the posteriorly inclined segment—are increased, and consequently, the “proclive” segment (the segment with the number of vertebrae inclined anteriorly) is decreased, so the total thoracic vertebrae number will always be 12.

The terms “declive” and “proclive” vertebra in kyphosis were introduced by Prof. RG Burwell 31 years ago [18,19,20].

The authors also write: “Based on previous findings and the results presented, we agree that hypokyphosis is not likely the primary causal factor in idiopathic scoliosis, rather a passive result of the combined effect of rotation and anterior opening of the apical intervertebral disc spaces [21]”. We agree with this fact—that hypokyphosis is not likely the primary causal factor—and we mention the following in the publication: “Regarding the role of hypokyphosis and its importance in IS aetiology, we stated earlier that the minor hypokyphosis of the thoracic spine and its minimal differences observed in the small curves studied compared with that of non-scoliotics, this adds to the view that the reduced kyphosis, by facilitating axial rotation, could be viewed as being permissive, rather than as aetiological, in the pathogenesis of IS [17]”. In other words, a straight (not bended) beam is more easily rotated than a curved one.

The authors next write: “However, the individual posteriorly inclined spine, specifically the relative length and inclination of that segment, is hypothesized to be a risk factor for scoliosis development and supported by recently published prospective data [22]”.

For our response, see our statement above.

In contrast to the proposition that sagittal alignment has no place in the aetiology of IS, we mention in our article that the minor hypokyphosis of the thoracic spine and its minimal differences observed in the small curves studied compared with that of non-scoliotics adds to the view that the reduced kyphosis, by facilitating axial rotation, could be viewed as being permissive, rather than as aetiological, in the pathogenesis of idiopathic scoliosis [17].

In conclusion, we would honestly like to thank the authors for their kindness in dealing with our article. With their comments, we were given the great opportunity to productively and positively expand and augment our arguments and discussion on such an interesting topic as that of the sagittal profile in relation to the aetiology of IS.

We are looking forward to future research contributions from other groups studying the aetiology of IS and further insights about the effect of the sagittal profile. Since the topic is very much under discussion, we might think about proceeding with the study and including more patients to increase its power. If contact to the patients is feasible, a follow-up might be possible, e.g., after growth completion.
